# AHR signaling is induced by infection with coronaviruses

**DOI:** 10.1038/s41467-021-25412-x

**Published:** 2021-08-26

**Authors:** Federico Giovannoni, Zhaorong Li, Federico Remes-Lenicov, María E. Dávola, Mercedes Elizalde, Ana Paletta, Ali A. Ashkar, Karen L. Mossman, Andrea V. Dugour, Juan M. Figueroa, Andrea A. Barquero, Ana Ceballos, Cybele C. Garcia, Francisco J. Quintana

**Affiliations:** 1 Ann Romney Center for Neurologic Diseases, Brigham and Women’s Hospital, Harvard Medical School, Boston, MA USA; 2 Instituto de Investigaciones Biomédicas en Retrovirus y SIDA (INBIRS), Universidad de Buenos Aires, Buenos Aires, Argentina; 3 Department of Pathology and Molecular Medicine, McMaster Immunology Research Centre, Michael DeGroote Institute for Infectious Disease Research, McMaster University, Hamilton, ON Canada; 4 Instituto de Ciencia y Tecnología Dr. Cesar Milstein (Consejo Nacional de Investigaciones Científicas y Técnicas-Fundacion Cassara), Buenos Aires, Argentina; 5 Laboratorio de Virología, Departamento de Química Biológica, Facultad de Ciencias Exactas y Naturales, Universidad de Buenos Aires. CONICET- Instituto de Química Biológica (IQUIBICEN), Buenos Aires, Argentina; 6 Laboratorio de Estrategias Antivirales, Departamento de Química Biológica, Facultad de Ciencias Exactas y Naturales, Universidad de Buenos Aires. CONICET- Instituto de Química Biológica (IQUIBICEN), Buenos Aires, Argentina; 7 Broad Institute of MIT and Harvard, Cambridge, MA USA

**Keywords:** Infectious diseases, Medical research

## Abstract

Coronavirus infection in humans is usually associated to respiratory tract illnesses, ranging in severity from mild to life-threatening respiratory failure. The aryl hydrocarbon receptor (AHR) was recently identified as a host factor for Zika and dengue viruses; AHR antagonists boost antiviral immunity, decrease viral titers and ameliorate Zika-induced pathology in vivo. Here we report that AHR is activated by infection with different coronaviruses, potentially impacting antiviral immunity and lung epithelial cells. Indeed, the analysis of single-cell RNA-seq from lung tissue detected increased expression of *AHR* and AHR transcriptional targets, suggesting AHR signaling activation in SARS-CoV-2-infected epithelial cells from COVID-19 patients. Moreover, we detected an association between *AHR* expression and viral load in SARS-CoV-2 infected patients. Finally, we found that the pharmacological inhibition of AHR suppressed the replication in vitro of one of the causative agents of the common cold, HCoV-229E, and the causative agent of the COVID-19 pandemic, SARS-CoV-2. Taken together, these findings suggest that AHR activation is a common strategy used by coronaviruses to evade antiviral immunity and promote viral replication, which may also contribute to lung pathology. Future studies should further evaluate the potential of AHR as a target for host-directed antiviral therapy.

## Abstract (web summary)

RNA viruses, such as Zika and Dengue, activate the aryl hydrocarbon receptor (AHR) signaling to restrict the antiviral response. Here, the authors report that coronaviruses also activate AHR signaling, potentially allowing them to escape the immune response, and show that AHR antagonism limits SARS-CoV-2 replication in vitro, suggesting that AHR could be targeted for antiviral therapy.

## Introduction

Coronaviruses (CoVs) are positive sense single-stranded RNA viruses of major agricultural and public health importance^1^. CoVs were considered of low risk to humans until 2002, when a severe acute respiratory syndrome (SARS) outbreak occurred in Guangdong, China^2–5^. Ten years later, the highly pathogenic Middle East respiratory syndrome coronavirus (MERS-CoV) emerged in Saudi Arabia^6^. In December 2019, an epidemic of coronavirus disease 2019 (COVID-19) caused by a severe acute respiratory syndrome coronavirus 2 (SARS-CoV-2) originated in Wuhan, China^7,8^. In most cases, SARS-CoV-2 causes asymptomatic or mild disease. The common symptoms of SARS-CoV-2 infection at onset are fever, fatigue, dry cough, myalgia, anosmia, and dyspnea. However, in 5-15 % of infected patients, a severe form of the disease causes life-threatening progressive respiratory failure^8–10^. In addition, the basic reproductive rate (R_0_) of SARS-CoV-2 was estimated to be higher than previous SARS-CoV-1 and MERS-CoV outbreaks as well as the 2009 influenza A H1N1 pandemic^11^. The virulence and high transmissibility of SARS-CoV-2 translate into a high number of patients needing intensive care support, putting stress on national health systems around the world. As of today, no specific therapeutic agents are available to treat COVID-19. Thus, there is an urgent unmet clinical need for candidate targets to treat and prevent SARS-CoV-2 infection.

The ligand-activated transcription factor aryl hydrocarbon receptor (AHR) controls multiple aspects of the immune response^12,13^. AHR activation by metabolites produced by tumors^14–16^ or in the context of viral infection^17^ interferes with the generation of protective immunity. Indeed, AHR suppresses the production of type I interferons (IFN-I)^18,19^, probably as part of a negative feedback mechanism because IFN-I induces AHR expression^20^. We recently showed that AHR activation during infection with Zika or dengue virus suppresses IFN-I-dependent and IFN-I-independent antiviral innate and intrinsic immunity^18^. Most importantly, an AHR antagonist optimized for human use boosted antiviral immunity, interfered with viral replication and ameliorated multiple aspects of Zika congenital syndrome including microcephaly in animal models^18^, identifying AHR as a candidate target for therapeutic intervention. Based on these findings and the urgent need for SARS-CoV-2 therapy, we investigated the potential role of AHR in CoV infection. Here, we show that infection with different CoVs activates AHR signaling in vitro and also in COVID-19 patients. Furthermore, pharmacologic AHR blockade reduces CoVs replication in vitro, identifying AHR as a candidate target for antiviral therapy.

## Results

### AHR signaling is activated by multiple coronaviruses

Early studies used microarrays to analyze the transcriptional response to infection by multiple CoVs including SARS-CoV-1 and the human coronavirus 229E (HCoV-229E) associated with the common cold^21^. We re-analyzed these datasets and detected increased expression of the AHR transcriptional targets *CYP1A1* and *CYP1B1* in response to SARS-CoV-1 and HCoV-229E infection. Similar findings describing AHR activation were recently reported in the context of infection by the murine coronavirus (M-CoV) in vitro and in vivo^22^ (Table 1). Indeed, we also detected increased expression of AHR-pathway genes, suggesting increased AHR signaling, in available gene expression datasets of infection with M-CoV^23^, HCoV-229E^24^, MERS-CoV^25^, and SARS-CoV-1^22^ (Table 1).

**Table 1:** Summary of studies reporting an effect on the AHR pathway after infection with different members of the *Alphacoronavirus* and *Betacoronavirus* genus of the *Coronaviridae* family.

Host	Genus	Virus	Model	Cell type or organ	Effect observed on the AHR pathway	Assay	Ref.
Mouse	*Betacoronavirus*	M-CoV	In vitro	Bone marrow-derived macrophages	Upregulation of *AHR* and downstream effectors *AHRR*, *CYP1B1*, *IDO1* and *TIPARP*	RT-qPCR	^22^
Mouse	*Betacoronavirus*	M-CoV	In vitro	Bone marrow-derived dendritic cells	Upregulation of *AHR* and downstream effectors *AHRR*, *CYP1B1*, *IDO1* and *TIPARP*	RT-qPCR	^22^
Mouse	*Betacoronavirus*	M-CoV	In vivo	Liver (C57BL/6 mice)	Upregulation of *AHR* and downstream effectors *CYP1B1*, *TIPARP*, *AHRR*, *IDO1*, *IDO2*, *TDO2*	RT-qPCR	^22^
Mouse	*Betacoronavirus*	M-CoV	In vitro	Bone marrow-derived macrophages	Upregulation of *AHR* and downstream effectors. Predicted AHR signaling pathway activation	RNA-Seq	This study
Human	*Alphacoronavirus*	HCoV-229E	In vitro	Human hepatoma (Huh7)	Upregulation of AHR downstream effectors *CYP1A1* and *CYP1B1*	Microarray	^21^
Human	*Alphacoronavirus*	HCoV-229E	In vitro	Human lung adenocarcinoma (A549)	Predicted AHR signaling pathway activation	Microarray	This study
Human	*Betacoronavirus*	MERS-CoV	In vitro	Human lung adenocarcinoma (Calu-3)	Upregulation of *AHR* and downstream effectors. Predicted AHR signaling pathway activation	RNA-Seq	This study
Human	*Betacoronavirus*	SARS-CoV-1	In vitro	Human hepatoma (Huh7)	Upregulation of AHR downstream effectors *CYP1A1* and *CYP1B1*	Microarray	^21^

In-depth analyses of RNA-Seq data from M-CoV-infected macrophages detected the upregulation of *AHR* and AHR-pathway-related genes such as *IDO2*, *CYP1B1*, *AHRR,* and *TIPARP* (Supplementary Fig. 1a). Indeed, ingenuity pathway analysis (IPA) detected increased AHR signaling as well as the activation of cell signaling processes involved in the antiviral response, including pattern recognition receptors, NF-κB, JAK/Stat, apoptosis, PKR, IRF, TNFR2, mTOR, and IL-6 (Supplementary Fig. 1b, Supplementary Data 1). Finally, we performed IPA upstream regulator analysis to identify potential upstream transcriptional regulators that control gene expression upon M-CoV infection. This analysis identified AHR-ARNT as a candidate regulator of the transcriptional response to M-CoV infection (Supplementary Fig. 1c).

**Fig. 1:**
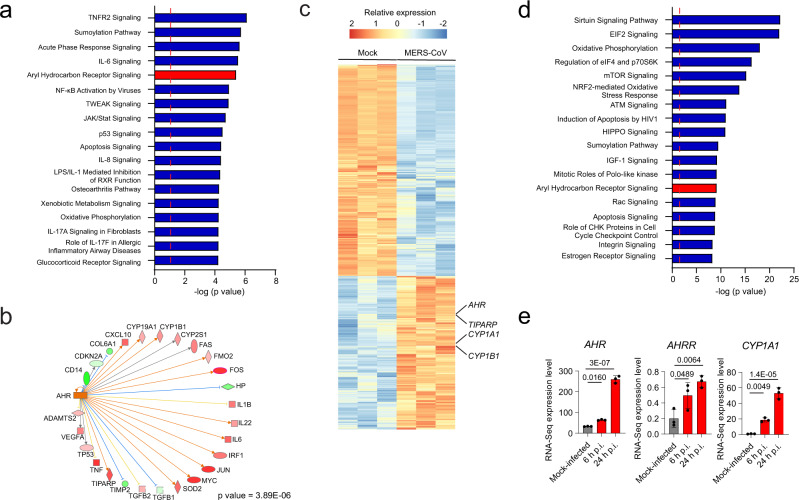
AHR signaling is triggered in response to infection with multiple CoVs. **a** IPA of pathways enriched in HCoV-229E-infected cells compared to mock-infected human lung adenocarcionma (A549) cells (*n* = 3 independent experiments per condition). Dashed red line indicates *p* = 0.05. *p* values were determined using a right-tailed Fisher’s exact test. **b** IPA Upstream regulator analysis identified AHR as a transcriptional regulator of the gene expression in response to HCoV-229E infection. *p* value was determined using a right-tailed Fisher’s exact test. Genes are represented as nodes. The shape of a node indicates the protein main function according to IPA. The color of the nodes represents expression levels: upregulated genes are shown in red and down-regulated genes are shown in green. The color of the lines indicates the predicted directional effect between two molecules. An orange line indicates a predicted upregulation, a blue line indicates a predicted downregulation and a yellow line indicates inconsistent findings. **c** Heatmap showing gene expression detected by RNA-seq analysis of mock-infected and MERS-CoV-infected human lung adenocarcinoma (Calu-3) cells (*n* = 3 independent experiments per condition). **d** IPA of pathways enriched in MERS-CoV-infected cells compared to mock-infected cells (*n* = 3 independent experiments per condition). Dashed red line indicates *p* = 0.05. *p* values were determined using a right-tailed Fisher’s exact test. **e** mRNA expression levels of *AHR*, *AHRR,* and *CYP1A1* determined at different times post-infection by RNA-Seq. Data represent the mean ± SD (*n* = 3 independent experiments). *p* values were determined by a one-way ANOVA followed by Dunnet’s post-hoc test. Source data are provided as a Source Data file. p.i.: post-infection.

Next, we analyzed a microarray-based dataset of HCoV-229E infected human lung adenocarcinoma epithelial cells (A549). Based on differential gene expression analysis, IPA predicted increased AHR signaling following HCoV-229E infection (Fig. 1a); AHR was also identified as a regulator of the transcriptional response to viral infection (Fig. 1b). We also detected the activation of additional pathways associated with the response to M-CoV, including TNFR2, IL-6, NF-κB, JAK/Stat, and apoptosis, suggesting that additional transcriptional responses, besides those driven by AHR, are shared by multiple CoVs (Fig. 1a, Supplementary Data 1). In support of this interpretation, the analysis of RNA-seq data from MERS-CoV infected human lung adenocarcinoma cells (Calu-3) detected the upregulation of *AHR* and AHR-pathway related genes (*CYP1A1*, *CYP1B*1, *TIPARP*) suggesting increased AHR signaling (Fig. 1c-e), alongside with additional pathways activated by other CoVs, including mTOR, apoptosis, and sumoylation (Fig. 1d, Supplementary Data 1). Taken together, these findings suggest that AHR signaling is activated during M-CoV, HCoV-229E, and MERS-CoV infection.

### AHR signaling is activated by SARS-CoV-2 infection in vitro

**Fig. 2:**
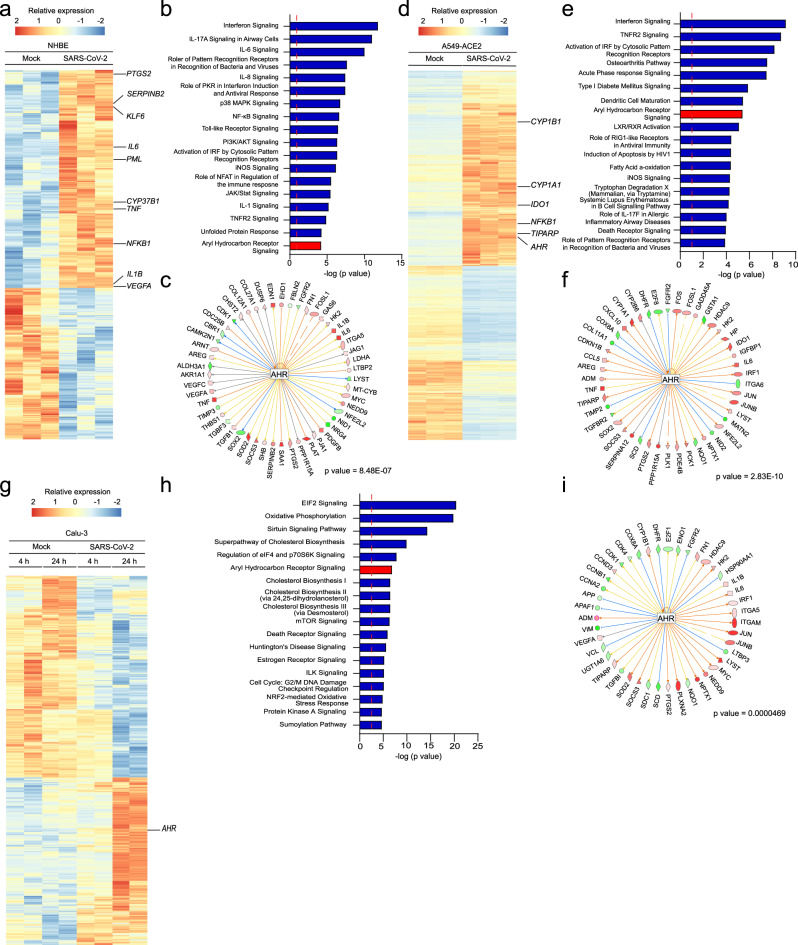
AHR signaling is triggered by SARS-CoV-2 infection. **a** Heatmap showing gene expression detected by RNA-seq analysis of mock-infected and SARS-CoV-2-infected primary human lung epithelium cells (*n* = 3 independent experiments per condition). **b** IPA of pathways enriched in SARS-CoV-2-infected primary human lung epithelium cells compared to mock-infected cells (*n* = 3 independent experiments per condition). Dashed red line indicates *p* = 0.05. *p* values were determined using a right-tailed Fisher’s exact test. **c** IPA Upstream regulator analysis identified AHR as an upstream transcriptional regulator of the gene expression in response to SARS-CoV-2 infection. *p* value was determined using a right-tailed Fisher’s exact test. Shapes and color coding as described in Fig. 1 (**b**). **d** Heatmap showing gene expression detected by RNA-seq analysis of mock-infected and SARS-CoV-2-infected A549-ACE2 cells (*n* = 3 independent experiments per condition). **e** IPA of pathways enriched in SARS-CoV-2-infected A549-ACE2 cells compared to mock-infected cells (*n* = 3 independent experiments per condition). Dashed red line indicates *p* = 0.05. *p* values were determined using a right-tailed Fisher’s exact test. **f** IPA Upstream regulator analysis identified AHR as an upstream transcriptional regulator of the gene expression in response to SARS-CoV-2 infection. *p* value was determined using a right-tailed Fisher’s exact test. Shapes and color coding as described in Fig. 1 (**b**). **g** Heatmap showing gene expression detected by RNA-seq analysis of mock-infected and SARS-CoV-2-infected Calu-3 cells at 4 and 24 h post-infection (*n* = 2 independent experiments per condition). **h** IPA of pathways enriched in SARS-CoV-2-infected Calu-3 cells compared to mock-infected cells at 24 h post-infection (*n* = 2 independent experiments per condition). Dashed red line indicates p = 0.05. p values were determined using a right-tailed Fisher’s exact test. **i** IPA Upstream regulator analysis identified AHR as an upstream transcriptional regulator of the gene expression in response to SARS-CoV-2 infection. *p* value was determined using a right-tailed Fisher’s exact test. Shapes and color coding as described in Fig. 1 (**b**).

SARS-CoV-2 is the causative agent of COVID-19^7,8^, thus we studied whether AHR signaling is activated after SARS-CoV-2 infection (Table 2). First, we analyzed RNA-seq data of SARS-CoV-2 infected and mock-infected normal human bronchial epithelial (NHBE) cells^26^. IPA analysis suggested increased AHR signaling in SARS-CoV-2 infected cells (Fig. 2a,b and Supplementary Data 1) together with other pathways previously associated with viral infection, either as part of the host immune response against viruses (interferon signaling^27–29^, IL-6^27,30–32^ IL-8^30,31^, NF-κB^33^, Toll-like receptor^29,34^, unfolded Protein Response^35^) or as part of a viral strategy to promote replication (PI3K/AKT^36^). Moreover, the analysis of upstream regulators identified AHR as a regulator of the transcriptional response of NHBE cells to SARS-CoV-2 infection (Fig. 2c). Taken together, these data suggest the involvement of AHR in the response to SARS-CoV-2 infection in NHBE cells.

**Table 2:** Summary of studies suggesting activation of AHR signaling after infection with SARS-CoV-2.

Host	Genus	Virus	Model	Cell type or organ	Effect observed on the AHR pathway	Assay	Ref.
Human	*Betacoronavirus*	SARS-CoV-2	In vitro	Primary human lung epithelium (NHBE)	Predicted AHR signaling pathway activation	RNA-Seq	This study
Human	*Betacoronavirus*	SARS-CoV-2	In vitro	Human lung adenocarcinoma (A549)-ACE2	Upregulation of *AHR* and downstream effectors. Predicted AHR signaling pathway activation	RNA-Seq	This study
Human	*Betacoronavirus*	SARS-CoV-2	In vitro	Human lung adenocarcinoma (Calu-3)	Upregulation of *AHR*. Predicted AHR signaling pathway activation	RNA-Seq	This study
Human	*Betacoronavirus*	SARS-CoV-2	Patients	Nasal swabs	Upregulation of *AHR* and *IDO*	RT-qPCR	This study

We then analyzed RNA-seq data of SARS-CoV-2-infected and control human lung adenocarcinoma (A549) cells overexpressing the viral receptor ACE2 (A549-ACE2)^26^. SARS-CoV-2 infection increased the expression of *AHR* and its transcriptional targets *CYP1A1* and *CYP1B1* among other genes (Fig. 2d). IPA analysis also suggested increased AHR signaling in SARS-CoV-2 infected cells, alongside other pathways known to participate in the antiviral response (Fig. 2e, Supplementary Data 1). In addition, upstream regulator analysis identified AHR as a regulator of the transcriptional response of A549-ACE2 cells to SARS-CoV-2 infection (Fig. 2f). Similarly, the RNA-Seq analysis of SARS-CoV-2-infected human lung adenocarcinoma Calu-3 cells detected the upregulation of *AHR* (Fig. 2g). IPA analysis suggested increased AHR signaling (Fig. 2h, Supplementary Data 1) following infection and identified AHR as a regulator of the transcriptional response (Fig. 2i). Taken together, these findings suggest that SARS-CoV-2 infection activates AHR signaling.

### Increased AHR signaling in SARS-CoV-2 infected patients

**Fig. 3:**
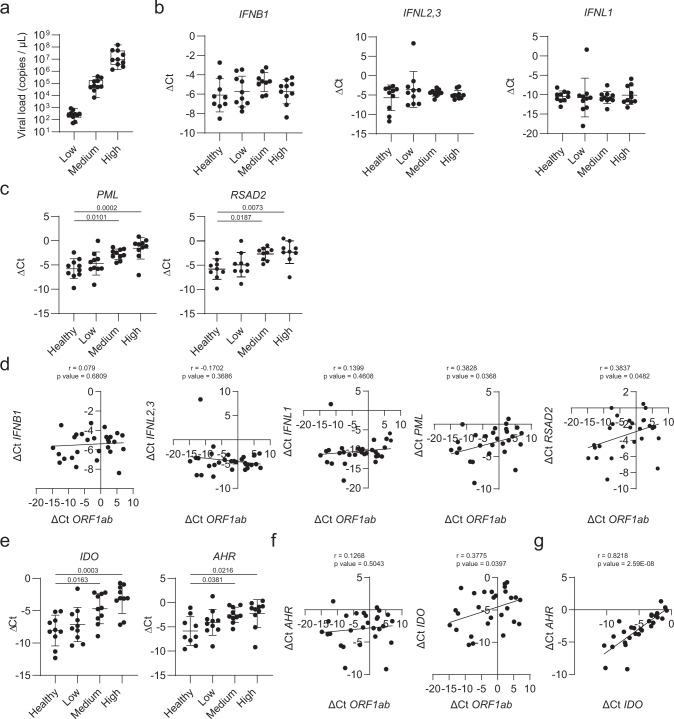
RT-qPCR analysis on nasal swabs from COVID-19 patients revealed activation of AHR signaling. **a** SARS-CoV-2 viral load in nasal swabs from COVID-19 patients was determined by RT-qPCR. Patients were classified intro three groups (low, medium, and high) on the basis of their viral load. Data is represented as a box and whiskers plot (*n* = 30 patients). Whiskers are plotted down to the minimum and up to the maximum value. The box extends from the 25th to 75th percentiles. The line in the middle of the box is plotted at the median. **b** *IFNB1, IFNL2,3*, and *IFNL1* mRNA expression were determined by RT-qPCR in nasal swab samples from healthy (*n* = 10) and COVID-19 patients (*n* = 30). Data represent the mean ± SD. *p* values were determined by a one-way ANOVA followed by Tukey’s post-hoc test. **c** *PML* and *RSAD2* mRNA expression was determined by RT-qPCR in nasal swab samples from healthy (*n* = 10) and COVID-19 patients (*n* = 30). Data represent the mean ± SD. *p* values were determined by a one-way ANOVA followed by Tukey’s post-hoc test. **d** Correlation analysis between expression levels of *IFNB1, IFNL2,3, IFNL1, PML, RSAD2,* and viral *ORF1ab* was computed using the Pearson correlation coefficient. Two-tailed *p* values were calculated. **e** *IDO* and *AHR* mRNA expression were determined as in (**b**). Data represent the mean ± SD. *p* values were determined by a one-way ANOVA followed by Tukey’s post-hoc test. **f** Correlation analysis between expression levels of *IDO, AHR,* and viral *ORF1ab* was calculated as in (**d**). Two-tailed p values were calculated. **g** Correlation analysis between *AHR* and *IDO* expression levels was calculated as in (**d**). Two-tailed *p* values were calculated. Source data are provided as a Source Data file.

To validate these findings we analyzed by RT-qPCR nasal swab samples collected from COVID-19 patients at the onset of clinical symptoms and controls. Based on the viral load, patient samples were classified into three groups: low, medium, and high (Fig. 3a). First, to briefly characterize the IFN-antiviral response, we analyzed the expression of *IFNL2,3*, *IFNL1, IFNB1,* and two well-known IFN-stimulated genes (ISGs) *PML* and *RSAD2*^37^ (Fig. 3b, c). We did not detect changes in *IFNL2,3*, *IFNL1, and IFNB1* expression, in agreement with reports of low IFN induction by SARS-CoV-2 infection^26,38–41^ and the role of several SARS-CoV-2 proteins as IFN-antagonists to limit antiviral response^42–44^. However, *PML* and *RSAD2* expression levels were significantly upregulated in COVID-19 patients bearing high and medium viral load. Indeed, *PML* and *RSAD2* expression showed a positive significant correlation with viral load (Fig. 3d).

AHR is a ligand-activated transcription factor. The enzyme indoleamine 2,3-dioxygenase (IDO) catalyzes the first and rate-limiting step of the generation of the AHR agonist kynurenine^12,14^. In addition, AHR activation is reported to promote *IDO* expression^45–47^. We found that the expression of *IDO* and *AHR* was upregulated in patients with medium and high viral load, in agreement with the increased AHR signaling detected in genome-wide transcriptional analyses (Fig. 3e, f). Moreover, we detected a positive correlation between *IDO* and *AHR* expression (Fig. 3g), which suggests that IDO upregulation in the context of SARS-CoV-2 infection may contribute to the activation of AHR signaling.

**Fig. 4:**
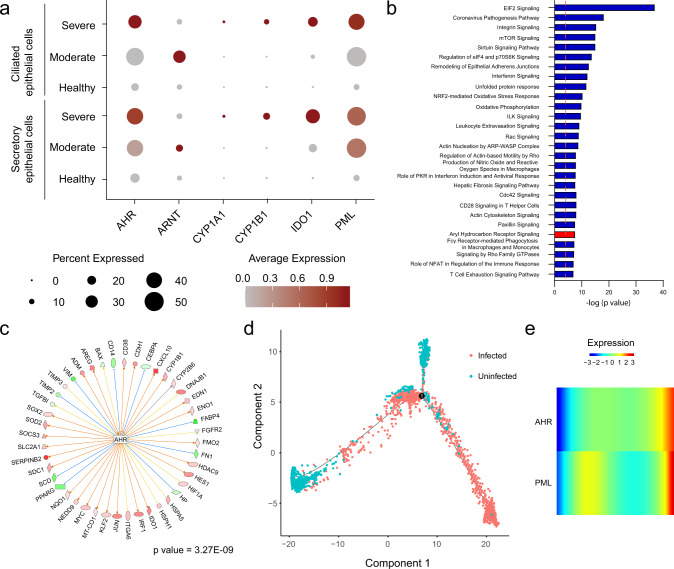
scRNA-Seq on BALF epithelial cells identified activation of AHR signaling in COVID-19 patients. **a***AHR*, *ARNT*, *CYP1A1, CYP1B1, IDO1,* and *PML* mRNA expression levels were determined by scRNA-Seq in ciliated and secretory epithelial cells from healthy or COVID-19 patients. **b** Ingenuity pathway analysis comparing uninfected to SARS-CoV-2 infected secretory epithelial cells. Dashed red line indicates *p* = 0.05. *p* values were determined using a right-tailed Fisher’s exact test. **c** Upstream regulator analysis on infected secretory epithelial cells identified AHR as a significant transcriptional regulator in the response of secretory epithelial cells to SARS-CoV-2 infection. *p* value was determined using a right-tailed Fisher’s exact test. **d** Cell fate trajectory analysis **e** Pseudo-time analysis of *AHR* and *PML* expression in infected cells.

To further investigate AHR signaling in the context of human SARS-CoV-2 infection, we analyzed a scRNA-Seq dataset of bronchoalveolar lavage fluid (BALF) cells from control and COVID-19 patients^48^. We identified BALF cell clusters corresponding to B cells, T cells (CD4+ and CD8+), macrophages/monocytes, NK cells, dendritic cells (mDC and pDC), plasma cells, and epithelial cells (ciliated and secretory) (Supplementary Fig. 2a, b). Next, we compared the mRNA expression levels of *AHR, ARNT, CYP1A1, CYP1B1, IDO1,* and *PML* in uninfected and SARS-CoV-2 infected ciliated and secretory epithelial cells (Fig. 4a). In agreement with our findings in infected cell lines and patient nasal swabs, we detected an increased expression of *AHR* and its target genes *CYP1A1* and *CYP1B1* in SARS-CoV-2-infected ciliated and secretory epithelial cells. Furthermore, IPA analysis in infected epithelial cells suggested the activation of pathways previously identified by bulk RNA-Seq, including IFN signaling, unfolded protein response, mTOR, PKR, sirtuin, and AHR signaling (Fig. 4b, Supplementary Data 1). Finally, upstream analysis identified AHR as a regulator of the transcriptional response of BALF epithelial cells to SARS-CoV-2 infection (Fig. 4c). In addition, a cell fate trajectory analysis (which uses gene expression to model the cell fate), showed that the uninfected cells and the infected cells spread at the end of the trajectory line (Fig.4d). *AHR* and *PML* expression are most highly expressed on the infected end of the trajectory, as shown by a pseudo-time analysis (Fig.4e). Taken together, these findings suggest that AHR signaling is upregulated in COVID-19 patients.

### AHR inhibition suppresses SARS-CoV-2 replication

**Fig. 5:**
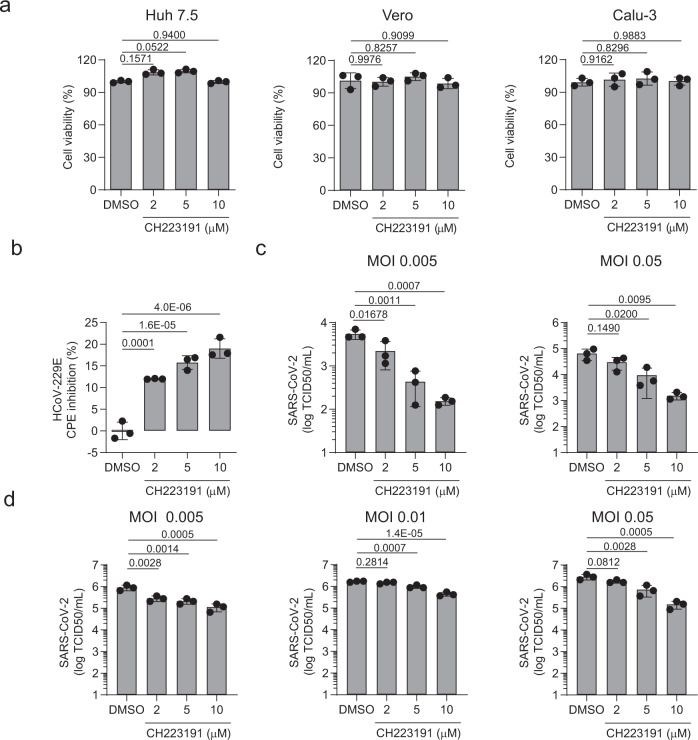
Pharmacological inhibition of AHR limits CoVs replication in vitro. **a** Huh 7.5, Vero, and Calu-3 cells were pretreated with the indicated concentrations of the AHR antagonist CH22319 and cell viability was determined using an MTS assay. Data represent the mean ± SD (*n* = 3 independent experiments). *p* values were determined by a one-way ANOVA followed by Tukey’s post-hoc test. **b** Huh 7.5 cells were pretreated with the indicated concentrations of the AHR antagonist CH223191 and infected with HCoV-229E (MOI = 0.1); 72 h p.i. CPE was quantified. Data represent the mean ± SD (n = 3 independent experiments). p values were determined by a one-way ANOVA followed by Tukey’s post-hoc test. **c** Calu-3 cells were pretreated with the indicated concentrations of CH223191 and infected with SARS-CoV-2 at the indicated MOI; 48 h p.i. supernatants were harvested for quantification of the viral titer. Data represent the mean ± SD (*n* = 3 independent experiments). *p* values were determined by a one-way ANOVA followed by Tukey’s post-hoc test. **d** Vero cells were used as described in (**c**). Data represent the mean ± SD (*n* = 3 independent experiments). *p* values were determined by a one-way ANOVA followed by Tukey’s post-hoc test. Source data are provided as a Source Data file.

We recently reported that AHR activation promotes the replication of Zika virus and dengue virus by interfering with cell intrinsic mechanisms and antiviral immunity^18^. Conversely, the pharmacologic inhibition of AHR suppressed the replication of Zika virus and dengue virus in vitro, and limited Zika virus replication and associated pathology in a pre-clinical animal model^18^. Hence, based on our finding of increased AHR signaling in response to infection with multiple CoVs, we investigated the effect of AHR inhibition by the AHR antagonist CH223191^49^ on the replication of HCoV-229E and SARS-CoV-2 in vitro. CH239131 had no effect on the viability of Huh 7.5, Vero, and Calu-3 cells (Fig.5a). However, AHR inhibition by CH223191 led to a dose-dependent reduction of HCoV-229E replication in Huh7.5 cells, as determined by the quantification of the cytopathic effect (CPE) (Fig.5b).

Next, we analyzed the effect of AHR inhibition by CH223191 on the replication of SARS-CoV-2 in Calu-3 and Vero cells and we found that AHR pharmacological inhibition limited SARS-CoV-2 replication in a wide range of MOI as determined by virus titration (Fig. 5c, d). Taken together, these findings suggest that AHR signaling promotes HCoV-229E and SARS-CoV-2 replication.

## Discussion

AHR has been identified as an important regulator of the immune response in autoimmunity, cancer, and infections^12,14^. Here we report the identification of AHR signaling as a common host response to infection by multiple CoVs. It has been reported that although some degree of NF-κB activation is needed for coronavirus replication, excessive NF-κB signaling may be deleterious for the virus^24^. Similarly, IFN-I is an important player in antiviral immunity, but excessive IFN-I production is linked to SARS-CoV-2 pathology in experimental models^50^. AHR limits NF-κB activation, and interferes with multiple antiviral immune mechanisms including IFN-I production and intrinsic immunity^18,19^. Hence, the findings reported in this manuscript suggest that, in addition to deficits in IFN-I-driven antiviral response driven by the genetic background or blocking antibodies in infected patients^51,52^, AHR signaling may contribute to the suppression of NF-κB- and IFN-I-driven antiviral immune mechanisms^18–20,53,54^. In this context, the activation of AHR signaling may represent a strategy exploited by CoVs to evade antiviral immunity and promote viral replication.

Our analyses suggested increased AHR signaling in SARS-CoV-2-infected lung epithelial cells, probably reflecting the upregulation of enzymes involved in the production of AHR agonists. Indeed, TDO and IDO2 expression is upregulated in response to viral infection^18^ as part of a mechanism that limits immunopathology^55^ but is exploited by pathogens to evade the immune response. Interestingly, AHR-deficient mice show enhanced repair of the lung bronchiolar epithelium following naphthalene injury^56^, concomitant with increased proliferation and earlier activation of basal cells involved in the replenishment of ciliated and secretory epithelial cells in multiple contexts including viral infection^57–59^. These findings suggest that AHR signaling may interfere with lung epithelial barrier integrity, contributing to the lung pathogenesis associated with SARS-CoV-1, MERS-CoV, and SARS-CoV-2 infection. Indeed, in a very recent study, SARS-CoV-2 infection was shown to trigger AHR signaling in lung epithelial cells leading to the overexpression of mucins -the major macromolecular components of mucus-, thickening the blood–air barrier and hindering O_2_ diffusion, directly contributing to lung pathology^60,61^.

Vaccines are a common approach to control viral infections, but this approach is not always successful^62–64^. An alternative approach is the development of antivirals, which have been shown to be clinically effective, for example for the treatment of human immunodeficiency virus (HIV)^65,66^ and hepatitis C virus (HCV)^67^ infection. Several drugs with antiviral activity against SARS-CoV-2 have been tested in vitro and in ongoing human studies^68^. However, viral-directed drugs such as lopinavir, ritonavir, and remdesivir seem to be ineffective in treating SARS-CoV-2 14 days after symptoms onset^69,70^, highlighting the need to identify additional therapeutic approaches. Host-directed antiviral therapy aims to target host factors that participate in virus replication. It has been postulated that this approach is less likely to select drug-resistant virus strains, although drug resistance against host-directed agents has been documented^71^. Most importantly, since host factors are usually shared by multiple viruses, antiviral drugs targeting a common host factor are expected to show a broader spectrum of action^72,73^. Based on its effects on the antiviral response^17–19^, AHR is an attractive candidate target for host-directed antiviral therapy. Indeed, using an in vitro approach, we showed that the AHR antagonist CH223191 reduced HCoV-229E and SARS-CoV-2 replication. Moreover, AHR antagonists have been recently shown to activate antiviral immunity, decrease viral titers and virus-induced pathology in the context of Zika and dengue virus infection^18^. Future studies are needed to further evaluate the potential of AHR antagonists for the treatment of infection by SARS-CoV-2 and other CoVs.

## Methods

### Whole-genome transcriptome profiling

All datasets analyzed were obtained from public repositories (Gene Expression Omnibus, GEO - NCBI). M-CoV-infected BMDMs dataset was accessed from at GSE144882. This dataset was generated from C57BL6 BMDMs that were infected with M-CoV (Strain A59) at a MOI of 1. 12 h p.i RNA was extracted for RNA-Seq. Mock-infected BMDMs were used as a control. Samples were obtained in triplicates^23^. HCoV-229E-infected A549 cells dataset was accessed at GSE89167. This dataset was generated from A549 cells that were infected with HCoV-229E at a MOI of 0.001. 6 h p.i. RNA was extracted for microarray analysis^24^. MERS-CoV-infected Calu-3 dataset was accessed at GSE139516. This dataset was generated from Calu-3 cells that were infected with MERS-CoV at a MOI of 4. 6 h or 24 h p.i. RNA was extracted for RNA-Seq analysis. Heatmap and IPA were obtained using the 24 h p.i. dataset. Mock-infected Calu-3 cells were used as a control. Samples were obtained in triplicates^25^. SARS-CoV-2-infected normal human bronchial epithelial cells (NHBE) dataset was accessed at GSE147507. This dataset was generated from NHBE cells that were infected with SARS-CoV-2 at a MOI of 2. 24 h p.i. total RNA was extracted for RNA-Seq analysis. Mock-infected NHBE were used as a control. Samples were obtained in triplicates^26^. SARS-CoV-2-infected A549 cells dataset was accessed at GSE147507. This dataset was generated from A549 cells overexpressing ACE2 that were infected with SARS-CoV-2 at a MOI of 2. 24 h p.i. total RNA was extracted for RNA-Seq analysis. Mock-infected A549 was used as a control. Samples were obtained in triplicates^26^. SARS-CoV-2 infected Calu-3 cells dataset was accessed at GSE148729. This dataset was generated from Calu-3 cells that were infected with SARS-CoV-2 at a MOI of 0.33. 4 and 24 h p.i. total RNA was extracted for RNA-Seq analysis. Mock-infected Calu-3 cells were used as control. Samples were obtained in duplicates^74^. SARS-CoV-2-infected and healthy patients’ BALF cells were accessed at GSE145926. BALF cells were obtained from healthy (*n* = 4) and COVID-19 patients (*n* = 9)^48^.

To perform RNA-sequencing analysis, the raw fastq files for all samples were downloaded and aligned to Human (GRCh38) and Mouse (GRCm38) reference genome using STAR v2.7.3a^75^. Then, the aligned reads were quantified using Rsem v1.3.1^76^. For differential expression analysis, the count matrix was built using the Rsem output for each sample, and then DESeq2^77^ was used to conduct differential expression analysis. The log2 fold change in the results was shrunk using ApeGlm^78^. Finally, differentially expressed genes were further analyzed using GSEA^79^ and IPA in order to find enriched pathways and upstream regulators.

### Clinical sample collection, SARS-CoV-2 viral load quantification, and gene expression analysis

Nasopharyngeal swabs were collected and deposited in 2 ml of saline solution at diverse hospitals and clinical centers in the Buenos Aires area, Argentina. These clinical samples were processed at the Instituto de Investigaciones Biomédicas en Retrovirus y SIDA (INBIRS, Buenos Aires, Argentina), a specialized center dedicated to SARS-CoV-2 diagnosis by RT-qPCR. The demographic and clinical characteristics of the patients are shown in Table 3. RNA was extracted from 300 µl of swab samples using a Chemagic 360-D automated extraction equipment (Perkin-Elmer). SARS-CoV-2 RNA was quantified using GeneFinder SARS-CoV-2 RT-PCR kit (Osang Health Care) which allows multiplex detection of viral genes N, E and RdRp and human gene RRP30. Viral load was quantified using Ct values of the *N* gene and a standard curve. After the determination of SARS-CoV-2, the remaining RNA was used for gene expression studies. cDNA was transcribed using the High-Capacity cDNA Reverse Transcription Kit (Life Technologies, 4368813). Gene expression was then measured by qPCR using SYBR Green I Master Mix (Roche). A list of primers used is provided (Supplementary Table 1).

**Table 3:** Demographic and clinical characteristics of patients with COVID-19.

Age, median (years)	31.6 (6–88)		
Male	11		
Female	19		
	All patients (*n* = 30)	Mild disease (*n* = 26)	Severe disease (*n* = 4)
	Symptoms		
Fever	50%	50%	50%
Cough	53%	54%	50%
Headache	27%	23%	50%
Diarrhea	3%	4%	0%
Odynophagia	23%	23%	25%
Respiratory rate per min > 25	7%	0%	50%
	Underlying conditions		
Hypertension	10%	8%	25%
Asthma	3%	0%	25%
Other	3%	0%	25%

For the purpose of this work, we used the remaining volume of anonymized samples that had been collected for clinical diagnosis of SARS-CoV-2 and therefore the IRB (Comite de Bioetica, Fundacion Huesped) deemed unnecessary to obtain informed consent from the patients. The authors were not involved in sample collection.

### Antiviral effect of pharmacological inhibition of AHR on CoVs replication in vitro

Drugs. CH223191 (Tocris) was resuspended in DMSO (Sigma) and used at a concentration ranging from 10 to 2 μM.

Cells. MRC5 cells were obtained from Dr. Vikram Misra (University of Saskatchewan, Canada). Huh 7.5 cells were received from Rodney Russell (Memorial University, Saint John’s, NL, Canada) with permission from C. Rice, Rockefeller University. Both cell lines were maintained in DMEM (Thermo Fisher) supplemented with 10 % fetal bovine serum (FBS) at 37 °C with 5% CO2.

Vero (C1008, clone E6, ATCC CRL-1586) and Calu-3 cells (ATCC, HTB­55) were cultured in DMEM supplemented with 5% heat-inactivated FBS (Thermo Fisher), penicillin (100 U/ml), and streptomycin (100 µg/ml).

Viruses and infections. HCoV-229E was a kind gift from Dr. Matthew Miller (McMaster University, Canada). HCoV-229E was propagated in MRC5 cells and virus titer was determined as 50% tissue culture infective doses (TCID50)^80^. Huh 7.5 cells were seeded in 96-well plates and inoculated with serial dilutions of virus stock for 1 h at 34 °C. Inoculum was removed and DMEM 3% FBS was added back. Plates were incubated for 5 days at 34 °C and, then, fixed with methanol and stained with Giemsa. For HCoV-229E CPE quantification, Huh 7.5 cells seeded in 24-well plates were pretreated with DMEM 3% FBS containing different concentrations of CH223191 for 2 h at 34 °C. After washing the cells with FBS-free media, cells were mock or infected with HCoV-229E at a MOI of 0.1 diluted in FBS-free media for 1 h at 34 °C. After removing the virus inoculum, cells were incubated with the same concentration of drug diluted in DMEM 3% FBS for 72 h at 34 °C. Cytopathic effect (CPE) as reduction of cellular viability was assessed using CellTiter 96® Aqueous One Solution Cell Proliferation Assay (MTS) (Promega), following the manufacturer’s instructions. Absorbance was measured using the SpectraMax i3 Multi-Mode Microplate Reader (Molecular Devices). Samples were analyzed in triplicate with a total of three independent experiments performed. % CPE inhibition was calculated relative to mock-infected cells and considering untreated infected cells as 0% inhibition.

SARS-CoV-2 was provided by Sandra Gallegos (Universidad Nacional de Córdoba, Argentina), propagated and titrated in Vero cells (2.85 × 10^6^ TCID50 per ml). For viral yield inhibition assays, Vero and Calu-3 cells were seeded in 96-well plates, pretreated with DMEM 2% FBS containing different concentrations of CH223191 (2, 5, and 10 µM) for 2 h at 37 °C and inoculated with SARS-CoV-2 at different MOIs (0.05, 0.01, and 0.005) for 1 h at 37 °C. Inoculum was removed and DMEM 2% FBS was added back. Plates were incubated for 2 days at 37 °C. Supernatants were collected and titrated in Vero cells and the infectious titer was expressed as TCID50 per ml. All work with infectious SARS-CoV-2 was done within biosafety cabinets in the biosafety level 3 facilities at INBIRS.

### Single-cell sequencing dataset processing and analysis

The single-cell RNA-Seq dataset of Bronchoalveolar lavage fluid from COVID patients and healthy controls was downloaded from the GEO repository GSE145926 and STAR v2.7.3a^75^ was used to align and quantify the gene expression for each cell in each dataset. Doublets were detected and removed using the Scrublet^81^ which utilizes the graphic-based clustering mechanism to detect cells with transcriptome profiles close to doublets. After the removal of doublets, the dataset was further filtered keeping only cells with more than 1000 UMIS, 500 genes detected and less than 75% of mitochondrial reads. In total 72,433 cells were kept in the dataset, with 25,588 of them from Healthy Control, 39.058 from Severe COVID patients and 7787 from Moderate COVID patients.

After the filtering process Seurat was used for normalization, batch effect correction, dimension reduction and clustering of the dataset^82^. The regularized negative binomial regression normalization method was used to normalize the data and regress out the effects of mitochondrial contents in cells^83^. Then the canonical pathway analysis built in Seurat was used to remove the batch effects between samples using the top 3000 variable genes. The UMAP and un-supervised clustering were done using the batch corrected data with top 75 principal components. Differential expression analysis was performed to identify upregulated gene markers for each cluster, which were used to identify cell types (Supplementary Fig. 2). The epithelial cells identified in the dataset were extracted for the differential expression comparing the infected cells against the uninfected cells. Monocle^84^ was used to conduct the pseudo time-series analysis of the epithelial cells. Genes that were significantly differentially expressed between different conditions were used to calculate the cell trajectory and pseudo time-series order. Differentially expressed genes were further analyzed using GSEA^79^ and IPA in order to find enriched pathways and upstream regulators.

### Statistical analysis

Microsoft Excel and GraphPad Prism software 8 were used for statistical analysis. For in vitro studies, biological samples were randomly allocated into experimental groups at the start of the experiment. No statistical methods were used to predetermine sample size, but our sample sizes are similar to those reported in previous publications. Investigators were not blinded for data collection and analysis. Blinding was not necessary because the results are quantitative and did not require subjective interpretation. Data distribution was assumed to be normal, but this was not formally tested. Differences between groups were tested as indicated in the figure legends. A *p*-value <0.05 was considered significant.

### Reporting summary

Further information on research design is available in the Nature Research Reporting Summary linked to this article.

## Supplementary information

Supplementary information

Description of Additional Supplementary Files

Supplementary Data 1

Reporting Summary

## Data Availability

The transcriptomics data used in this study are available in the NCBI GEO database (https://www.ncbi.nlm.nih.gov/geo/) under accession codes GSE144882, GSE89167, GSE139516, GSE148729, GSE147507, GSE145926. Human GRCh38.p13 [https://www.ncbi.nlm.nih.gov/assembly/GCF_000001405.39] and Mouse GRCm38.p6 [https://www.ncbi.nlm.nih.gov/assembly/GCF_000001635.26/] reference genomes were used in this study. Source data are provided with this paper.

## Source data

Source Data
