# Supplementary material for: AHR signaling is induced by infection with coronaviruses
Source: Nat Commun. 2021 Aug 26;12:5148. doi: 10.1038/s41467-021-25412-x (PMC8390748; doi:10.1038/s41467-021-25412-x)
Supplement: Supplementary file 1 — Supplementary information [file 41467_2021_25412_MOESM1_ESM.pdf]

Supplementary Information

AHR signaling is induced by infection with coronaviruses

Federico Giovannoni[1], Zhaorong Li[1], Federico Remes-Lenicov[2], María E. Dávola[3], Mercedes Elizalde[2], Ana Paletta[2], Ali A. Ashkar[3], Karen L. Mossman[3], Andrea V. Dugour[4], Juan M. Figueroa[4], Andrea A. Barquero[5], Ana Ceballos[2], Cybele C. Garcia[6,*] and Francisco J. Quintana[1,7,*]

[1] Ann Romney Center for Neurologic Diseases, Brigham and Women's Hospital, Harvard Medical School, Boston, MA 02115, USA

[2] Instituto de Investigaciones Biomédicas en Retrovirus y SIDA (INBIRS), Universidad de Buenos Aires, Buenos Aires, Argentina.

[3] Department of Pathology and Molecular Medicine, McMaster Immunology Research Centre, Michael DeGroote Institute for Infectious Disease Research, McMaster University, Hamilton, ON L8S 4K1, Canada

[4]Instituto de Ciencia y Tecnología Dr. Cesar Milstein (Consejo Nacional de Investigaciones Científicas y Técnicas-Fundacion Cassara)

[5] Laboratorio de Virología, Departamento de Química Biológica, Facultad de Ciencias Exactas y Naturales, Universidad de Buenos Aires. CONICET- Instituto de Química Biológica (IQUIBICEN), Buenos Aires, Argentina

[6] Laboratorio de Estrategias Antivirales, Departamento de Química Biológica, Facultad de Ciencias Exactas y Naturales, Universidad de Buenos Aires. CONICET- Instituto de Química Biológica (IQUIBICEN), Buenos Aires, Argentina

[7] Broad Institute of MIT and Harvard, Cambridge, MA, USA.

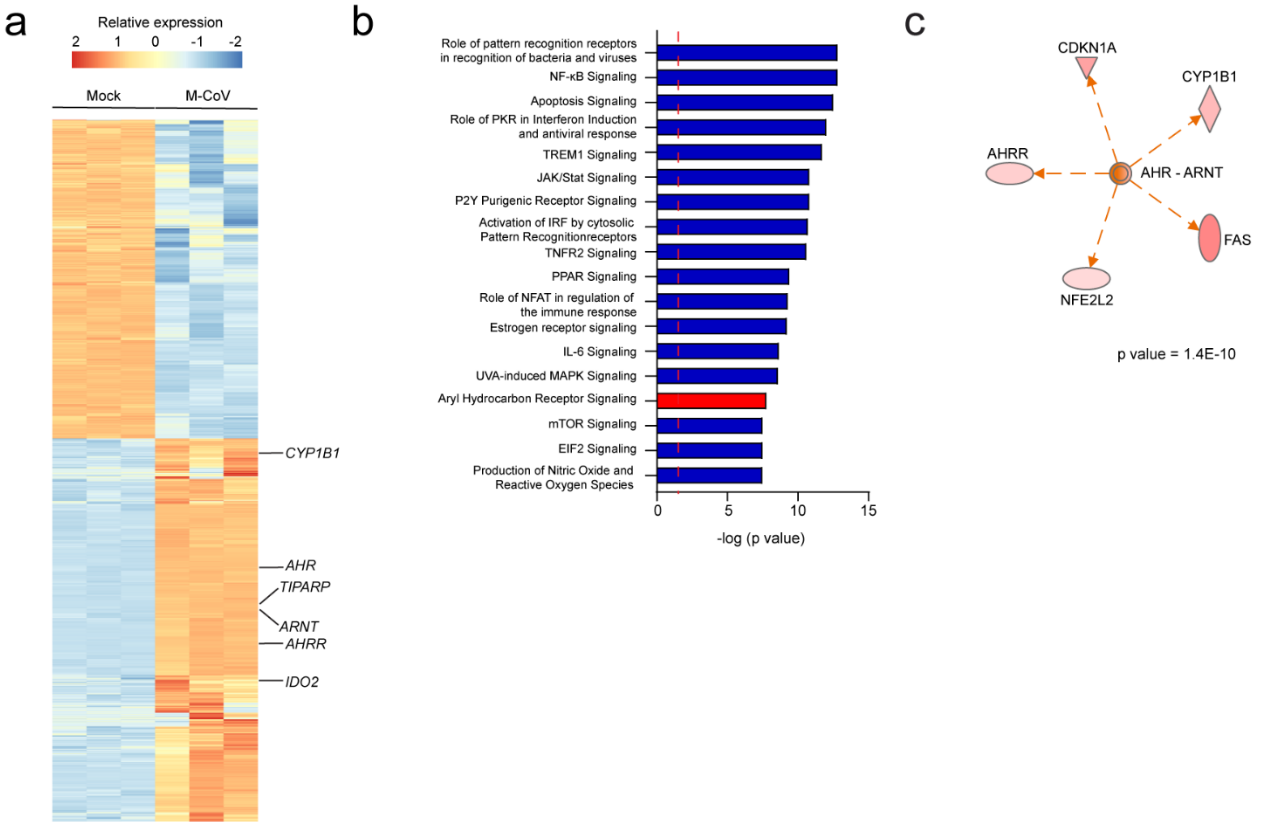

Supplementary Figure 1. RNA-Seq analyses of M-CoV-infected macrophages. (a) Heatmap showing gene expression levels detected by RNA-seq analysis of mock-infected and M-CoV-infected bone marrow-derived macrophages (n=3 independent experiments per condition) (b) IPA of pathways enriched in M-CoV-infected cells compared to mock-infected cells (n=3 independent experiments per condition). Dashed red line indicates p=0.05. p values were determined using a right-tailed Fisher's exact test. (c) IPA Upstream regulator analysis identified AHR as a transcriptional regulator of gene expression in response to M-CoV infection. p value was determined using a right-tailed Fisher's exact test.

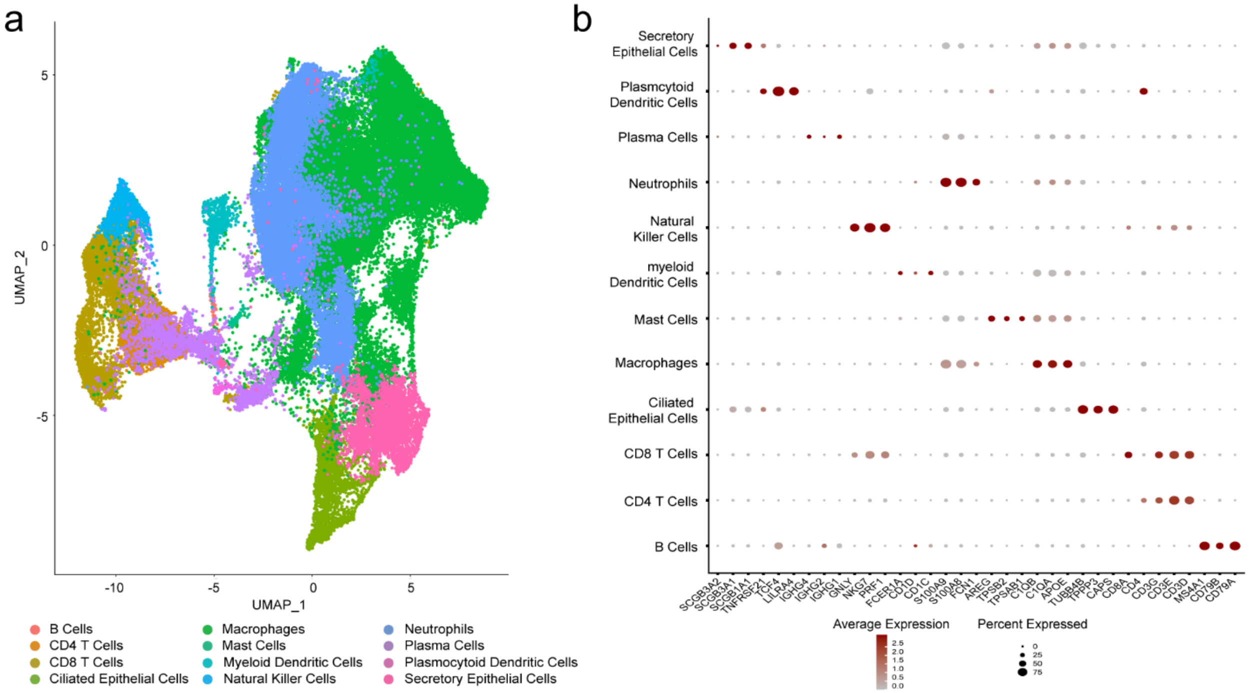

Supplementary Figure 2. scRNA-Seq on BALF cells identified 12 major cell types. (a) Uniform Manifold Approximation and Projection (UMAP) plot of BALF cells. (b) Dot plot of marker genes for each major cluster of cells identified. Dot size represents the percentage of cells in each cluster expressing a given gene. Color coding indicates average expression level for each gene.

Supplementary Table 1. **Primers used for RT-qPCR analysis.**

Gene	Sense	Sequence
IFNB1	Forward	TAGCACTGGCTGGAATGAGA
	Reverse	TCCTTGGCCTTCAGGTAATG
IFNL1	Forward	CTTCCAAGCCCACCACAACT
	Reverse	GGCCTCCAGGACCTTCAGC
IFNL2/3	Forward	GCCACATAGCCCAGTTCAAGTC
	Reverse	GGCATCTTTGGCCCTCTTAAA
IDO	Forward	GGGACACTTTGCTAAAGGCG
	Reverse	GTCTGATAGCTGGGGGTTGC
AHR	Forward	ACATCACCTACGCCAGTCGC
	Reverse	TCTATGCCGCTTGGAAGGAT
PML	Forward	TTCTGCTCCAACCCCAAC
	Reverse	CGCTGATGTCGCACTTGA
RSAD2	Forward	CGTGGAAGAGGACATGACGGAAC
	Reverse	CCGCTCTACCAAATCCAGCTTC
