# Supplementary material for: AHR signaling is induced by infection with coronaviruses
Source: Nat Commun. 2021 Aug 26;12:5148. doi: 10.1038/s41467-021-25412-x (PMC8390748; doi:10.1038/s41467-021-25412-x)
Supplement: Supplementary file 2 — Description of Additional Supplementary Files [file 41467_2021_25412_MOESM2_ESM.docx]

Description of Additional Supplementary Files

File Name: Supplementary Data 1

Description: Ingenuity pathway analysis (IPA) was performed comparing SARS-CoV-2-infected cells to mock-infected cells. p values were determined using a Fisher’s exact test.
