# Supplementary material for: AHR signaling is induced by infection with coronaviruses
Source: Nat Commun. 2021 Aug 26;12:5148. doi: 10.1038/s41467-021-25412-x (PMC8390748; doi:10.1038/s41467-021-25412-x)
Supplement: Supplementary file 4 — Reporting Summary [file 41467_2021_25412_MOESM4_ESM.pdf]

nature portfolio

Corresponding author(s): Francisco J. Quintana
Cybele C. Garcia

Last updated by author(s): Jul 1, 2021

Reporting Summary

Nature Portfolio wishes to improve the reproducibility of the work that we publish. This form provides structure for consistency and transparency in reporting. For further information on Nature Portfolio policies, see our Editorial Policies and the Editorial Policy Checklist.

Statistics

For all statistical analyses, confirm that the following items are present in the figure legend, table legend, main text, or Methods section.

n/a	Confirmed	
☐	☒	The exact sample size (*n*) for each experimental group/condition, given as a discrete number and unit of measurement
☐	☒	A statement on whether measurements were taken from distinct samples or whether the same sample was measured repeatedly
☐	☒	The statistical test(s) used AND whether they are one- or two-sided<br>*Only common tests should be described solely by name; describe more complex techniques in the Methods section.*
☐	☒	A description of all covariates tested
☐	☒	A description of any assumptions or corrections, such as tests of normality and adjustment for multiple comparisons
☐	☒	A full description of the statistical parameters including central tendency (e.g. means) or other basic estimates (e.g. regression coefficient) AND variation (e.g. standard deviation) or associated estimates of uncertainty (e.g. confidence intervals)
☐	☒	For null hypothesis testing, the test statistic (e.g. *F*, *t*, *r*) with confidence intervals, effect sizes, degrees of freedom and *P* value noted<br>*Give P values as exact values whenever suitable.*
☒	☐	For Bayesian analysis, information on the choice of priors and Markov chain Monte Carlo settings
☒	☐	For hierarchical and complex designs, identification of the appropriate level for tests and full reporting of outcomes
☐	☒	Estimates of effect sizes (e.g. Cohen's *d*, Pearson's *r*), indicating how they were calculated

Our web collection on statistics for biologists contains articles on many of the points above.

Software and code

Policy information about availability of computer code

Data collection	No software was used for collecting data
Data analysis	Microsoft Excel 2016<br>Graphpad Prism 8<br>STAR (v2.7.3a)<br>Rsem v1.3.1<br>DESeq2 (v 1.24.0)<br>Apeglm 1.8.0<br>GSEA (v4.0.0)<br>IPA (v 1-13, Qiagen)<br>Seurat (3.0)<br>MAST (5.0.5)<br>R (3.3.3)<br>Monocle (v 2.9.0)

For manuscripts utilizing custom algorithms or software that are central to the research but not yet described in published literature, software must be made available to editors and reviewers. We strongly encourage code deposition in a community repository (e.g. GitHub). See the Nature Portfolio guidelines for submitting code & software for further information.

Data

Policy information about availability of data

All manuscripts must include a data availability statement. This statement should provide the following information, where applicable:

- Accession codes, unique identifiers, or web links for publicly available datasets
- A description of any restrictions on data availability
- For clinical datasets or third party data, please ensure that the statement adheres to our policy

The authors declare that the raw data supporting the findings of this study are publicly available. Transcriptomics data (RNA-Seq or microarray) from virus-infected samples, including M-CoV, HCoV-229E, and MERS-CoV were accessed at GSE144882, GSE89167, and GSE139516, respectively. SARS-CoV-2 transcriptomics data were accessed at GSE148729 and GSE147507. SARS-CoV-2 scRNA-Seq data was accessed at GSE145926. Human (GRCh38.p13) and Mouse (GRCm38.p6) reference genomes were used in this study.

Field-specific reporting

Please select the one below that is the best fit for your research. If you are not sure, read the appropriate sections before making your selection.

☒ Life sciences ☐ Behavioural & social sciences ☐ Ecological, evolutionary & environmental sciences

For a reference copy of the document with all sections, see nature.com/documents/nr-reporting-summary-flat.pdf

Life sciences study design

All studies must disclose on these points even when the disclosure is negative.

Sample size	No statistical methods were used to pre-determine sample sizes but they are similar to those reported in previous publications. Giovannoni et al, Nat Neuro, 2020 ; Riva et al, Nature, 2020.
Data exclusions	No data was excluded
Replication	For RNA-Seq, 3 independent experiments (n=3) per condition (mock-infected or virus-infected) were used for analysis.<br>For In vitro experiments (cell viability, antiviral assays), 3 independent experiments were performed.<br>Replication information for each experiment is indicated in figure legends
Randomization	Not applicable for transcriptomics studies because they were previously designed and performed by other groups. We used the publicly available data deposited on the Gene Expression Omnibus database (NCBI).<br>For in vitro experiments, samples were allocated randomly into experimental groups at the start of each individual experiment.
Blinding	Investigators were not blinded for data collection and analysis. Blinding was not necessary because the results are quantitative and did not require subjective interpretation.

Reporting for specific materials, systems and methods

We require information from authors about some types of materials, experimental systems and methods used in many studies. Here, indicate whether each material, system or method listed is relevant to your study. If you are not sure if a list item applies to your research, read the appropriate section before selecting a response.

Materials & experimental systems

n/a	Involved in the study	
☒	☐	Antibodies
☐	☒	Eukaryotic cell lines
☒	☐	Palaeontology and archaeology
☒	☐	Animals and other organisms
☐	☒	Human research participants
☒	☐	Clinical data
☒	☐	Dual use research of concern

Methods

n/a	Involved in the study	
☒	☐	ChIP-seq
☒	☐	Flow cytometry
☒	☐	MRI-based neuroimaging

Eukaryotic cell lines

Policy information about cell lines

Cell line source(s)	Vero (ATCC, CCL-81)<br>Calu-3 (ATCC, HTB55)

	MRC5 (ATCC, CCL-171) were obtained from Dr. Vikram Misra (University of Saskatchewan, Canada).<br>Huh 7.5 cells were received from Rodney Russell (Memorial University, Saint John's, NL, Canada) with permission from C. Rice, Rockefeller University
Authentication	None of the cell lines used were authenticated
Mycoplasma contamination	Cell lines tested negative for Mycoplasma contamination
Commonly misidentified lines<br>(See ICLAC register)	No commonly misidentified cell lines were used in the study

Human research participants

Policy information about studies involving human research participants

Population characteristics	The sample is representative as it includes all sexes, ages without any selection other than the fact that the patients tested positive for COVID-19 by qPCR.
Recruitment	Nasopharyngeal swabs were collected and deposited in 2 ml of saline solution at diverse hospital and clinical centers at the Buenos Aires area, Argentina. These clinical samples were processed at the Instituto de Investigaciones Biomédicas en Retrovirus y SIDA (INBIRS, Buenos Aires, Argentina), a specialized center dedicated to SARS-CoV-2 diagnosis by RT-qPCR. Samples were not self-selected, avoiding potential self-selection bias.
Ethics oversight	For the purpose of this work, we used the remaining volume of anonymized samples that had been collected for clinical diagnosis of SARS-CoV-2 and therefore the IRB (Comite de Bioetica, Fundacion Huesped) deemed unnecessary to obtain informed consent from the patients.

Note that full information on the approval of the study protocol must also be provided in the manuscript.
